# The effects of amalgam contamination and different surface modifications on microleakage of dentin bonded to bulk fill composite when using different adhesive protocols

**DOI:** 10.1186/s12903-022-02214-1

**Published:** 2022-05-18

**Authors:** Nojoud Alshehri, Abdullah Aljamhan, Mohammed Bin-Shuwaish

**Affiliations:** Restorative Dental Sciences Department, College of Dentistry, King Saud University, Riyadh, Kingdom of Saudi Arabia

**Keywords:** Chlorhexidine, Dentin refreshment, Resin-based composite, Universal adhesive system

## Abstract

**Background:**

To evaluate the effect of amalgam contamination, different surface treatments, and adhesive protocols on dentin microleakage to bulk-fill composite resin material.

**Methods:**

Forty teeth were fixed in (polyvinyl siloxane) PVS molds, and the Class II cavities were placed on mesial and distal aspects. Thirty teeth were restored by amalgam and thermocycled to 10,000 cycles (5 and 55 °C, 30-s dwell time). The rest were restored with Filtek one Bulk Fill composite without amalgam predecessor. Samples were divided into: G1 (dentin pretreated with 2% chlorhexidine gluconate), G2 (0.5 mm of dentin was removed), G3 (no surface modification), and G4 (control, where composite was bonded to sound dentin without amalgam predecessor.). Single Bond Universal Adhesive system was used to bond the composite material, by using the etch-and-rinse protocol in the mesial cavity preparation and self-etch protocol in the distal. Specimens underwent thermocycling for 5000 cycles, then embedded in silver nitrate and sectioned for stereomicroscope examination. Descriptive statistics, Mann–Whitney U test, and Kruskal–Wallis test were used to analyze the results at *p* < 0.05.

**Results:**

The highest microleakage score values (4.00) were found in the G2, and G4 in etch-and-rinse protocol. While the lowest scores were found in G2 when using self-etching protocol (1.5). Lower microleakage values were associated with the chlorhexidine treatment group for both adhesive protocols. No significant differences were found between amalgam contaminated and non-contaminated groups.

**Conclusions:**

Amalgam contamination did not affect microleakage. Self-etching adhesive protocol significantly reduced microleakage for all groups irrespective of the surface treatment. Chlorhexidine pretreatment improved microleakage for both adhesive protocols but had no significant effect.

## Background

Amalgam has served as the primary material for direct tooth restoration for many decades. Despite its great durability in the harsh oral environment and its great clinical success [1], there has been a trend toward replacing amalgam with esthetic restorations, due to material failure, claimed health hazards, or esthetic reasons [2]. Although, In 2013, The Minamata Convention agreed to phase down amalgam on environment to “protect the human health and the environment from anthropogenic emissions and releases of mercury and mercury compounds” [3], however, the World Health Organisation (WHO), World Dental Federation (FDI), International Association for Dental Research (IADR) and other organizations recommended gradual reduction in the use of dental amalgam [4]. When these amalgam restorations are decided to be replaced by esthetic restorations, theses successor esthetic restorations may be bonded directly or indirectly to the prepared cavity following removal of amalgam restorations. In both conditions, the creation of a good bond is important for the success of such restorations [5].

Although there has been a great improvement in adhesive technology, many problems still exist when bonding esthetic restorations to dentin substrate. These include gap formation and microleakage [6], which may increase the risk of recurrent caries, staining, or postoperative sensitivity [7]. Many factors affect the bond quality: the bonding protocol, the presence of contamination, the bonded substrate, and surface preparation of the tooth [8].

One major factor that leads to microleakage is the polymerization shrinkage of composite restoration, which results in stresses exceeding the bond strength of adhesive [9] leading to gap formation in the interface between the tooth structure and the filling materials. This in turn results in bacterial and fluid penetration, and, consequently, marginal discoloration [10]. This problem is magnified when bonding to dentin substrate, which contains less mineral content than enamel. Therefore, deep class II restorations might be more susceptible to recurrent caries [11].

Many solutions were introduced to improve the bond quality of the dentinal structure. These have included different surface treatments (e.g., use of matrix metalloproteinase inhibitors, like chlorhexidine digluconate) [12], different adhesive protocols [13], the use of low shrinking and bulk fills composites, and the layering of conventional composites [14]. Despite attempts to improve the marginal adaptation and reduce polymerization shrinkage, the achievement of perfect seal and prevention of marginal leakage is very challenging [14].

Nonetheless, many of these factors that affect dentin bond quality to composite restorations have been mostly studied in sound dentin, but not dentin that was contaminated with amalgam products. Therefore, more clinical and laboratory details on the effects of these factors on bonded esthetic restorations replacing amalgam fillings are required.

This study aims to evaluate the microleakage of Class II composite restorations bonded to amalgam-contaminated dentin and the effects of two surface treatments: 2% chlorhexidine and the removal of 0.5 mm of contaminated dentin using two adhesive protocols, with a primary null hypothesis; that there would be no significant difference between amalgam- and non-contaminated dentin bonded to bulk-fill composite using different surface treatments, and a secondary hypothesis; that there would be no significant difference in microleakage between different universal adhesive system protocols within each treatment group.

## Methods

### Sample size determination

The sample size was calculated using Basic Functions for Power Analysis 1.3-0 [15]. The sample size was determined to detect an effect size of 0.56-unit difference among four comparison groups; the required number of samples in each group was 9.5 (n = 10) at alpha set to 0.05 and power = 0.80.

### Materials

Table 1 summarizes the materials used in this study. High Copper amalgam (Futura Standard, Ardent, Arlandastad, Sweden), Filtek one Bulk fill restorative (A1 shade) (3 M ESPE, St. Paul, MN, USA) composite resin, one universal adhesive system (Single Bond Universal Adhesive, 3 M ESPE, St. Paul, MN, USA). Two surface treatments will be employed in this study: the first is the use of 2% chlorhexidine digluconate (Consepsis Cavity Cleanser, Bisco Inc., Schaumburg, IL, USA), and the second is the removal of 0.5 mm of stained dentin.

**Table 1:** Materials used in the study

Material	Company	Composition
Filtek™ One Bulk-Fill Posterior Composite Resin Restorative Material Shade A1	3 M ESPE, St. Paul, MN, USA	AFM, AUDMA, UDMA, and 1, 12-DDMA
		Fillers: combination of a 20-nm silica filler, 4- to 11-nm zirconia filler, and an ytterbium trifluoride filler
		Inorganic filler: 76.5% by weight (58.5% by volume)
3 M™ Single Bond Universal Adhesive Bonding System	3 M ESPE, St. Paul, MN, USA	MDP monomer, HEMA, ethanol, vitrebond copolymer, filler, water, initiators, dimethacrylate resins, and silane
Scotchbond™ Universal Etchant Phosphoric Acid	3 M ESPE, St. Paul, MN, USA	32% Phosphoric acid in water, thickening agent, and colorants
Consepsis® Antibacterial Solution (chlorhexidine)	Ultradent, South Jordan, UT, USA	2.0% Chlorhexidine gluconate solution
Ardent Futura Standard ® High Copper Amalgam Restorative Material	Ardent, Arlandastad, Sweden	50% Mercury
		50% Alloy lathe cut powder: 44.5% silver, 30% tin, 25.5% copper

### Light curing protocol of resin composite

A light-emitting diode curing unit was used to cure the composite resin and adhesive system. The curing unit (Bluephase G2, ivoclar vivadent, Schaan, Liechtenstein) is a poly wave emitting unit with a light intensity of 1200 mW/cm^2^ for 20 s producing light in the range of 385–515 nm wavelength. The light tip was kept perpendicular and 1 mm away from the sample. After the curing of all five samples, a radiometer (Demetron L.E.D Radiometer, Kerr, Detroit, Michigan, USA) was used to check light intensity to ensure sufficient energy.

### Adhesive protocols

Two adhesion protocols were used for the selected adhesive bonding system:

- *Etch-and-rinse mode (ER)* On the mesial cavity preparations, dentin was acid-etched with 32% phosphoric acid (Scotchbond Universal Etchant, 3 M ESPE, St. Paul, MN, USA) for 15 s and washed for 20 s then air-dried. Using a micro-brush, the adhesive was rubbed into the dentin surface for 20 s, then air-dried for five seconds to evaporate the solvent, and light-cured for 10 s.
- *Self-etch mode (SE)* On the distal cavity preparations, the same steps were followed as in the etch-and-rinse mode, except for phosphoric acid etching.

Sample selection and preparation:

Forty extracted non-carious human molar teeth were collected from different clinics (private and governmental). Teeth with caries, previous restorations, cracks, stains, or root-canal treated were excluded from the study. All teeth were cleaned and stored in 0.25% thymol at refrigerator temperature (at 4 °C) for two weeks until used.

Teeth were fixed in polyvinyl-siloxane putty materials for support during cavity preparation and matrix placement during restorations.

Two class II box cavity preparations—one on the mesial and the other on distal surfaces—were conducted on each tooth. The dimensions of the preparations were 3 mm in pulpal depth and 4 mm in width, with the gingival margin located in the dentin about 1 mm below the cemento-enamel junction (CEJ). Teeth were randomly subdivided into four groups, 10 teeth per each group (n = 10). In the first three groups, teeth were restored with amalgam restorations, then were thermocycled for 10,000 cycles to simulate one year of clinical service [16] in a water bath between 5 and 55 °C with 30 s dwell time in between.

After retrieving each sample from the water bath, the amalgam was carefully removed, and teeth were then restored with composite restorations according to the manufacturer’s instructions using etch-and-rinse or self-etch adhesive protocols.

### Study groups

(G1: CHX) Composite restorations were bonded to a dentin surface that was previously contaminated with amalgam and pretreated with 2% chlorhexidine gluconate.

(G2: DR) Composite restorations were bonded to dentin surface that was previously contaminated with amalgam with additional removal of 0.5 mm of dentin margins.

(G3: NT) Composite restorations were bonded to a dentin surface that was previously contaminated with amalgam without any surface treatment.

(G4: Control) Composite restorations were bonded to sound dentin surface (no surface treatment and without amalgam predecessor).

All teeth were then stored in distilled water at room temperature for 24 h for polymerization reaction and thermo-cycled for 5000 cycles (5 °C/55°C) with a dwell time of 30 s and a transfer time of 10 s.

### Evaluation of microleakage

The tooth samples were coated with two layers of acid-resistant varnish to about 1 mm away from the preparation margins. Specimens were immersed in the prepared 50% ammoniacal silver nitrate (pH = 9.5) solution for 24 h in the dark [17]. Specimens were then thoroughly rinsed in distilled water for five minutes and immersed in a photo-developer solution for 12 h under fluorescent light to reduce the silver ions to metallic silver [18]. After removal from the developing solution, the specimens were placed under running water for five minutes.

The tooth samples were sectioned mesio-distally into three longitudinal slices [19] using a precision circular saw with a 0.2 mm thickness (ISOMET 1000-BUEHLER, Illinois, USA) under water cooling. Sectioned samples were arranged accordingly in a mold for epoxy resin material to securely hold the samples. The secured samples were then polished with 400, 600, 1200, and 2000 grit silicon-carbide paper under constant water cooling. After rinsing, samples were immersed in 37% phosphoric acid for 30 s, then immersed in 5% sodium hypochlorite (NaOCL) for 30 min. Finally, they were agitated in an ultrasonic cleaner (L & R Ultrasonics) for five minutes to remove the smear layer [20].

Dye penetration in each cavity and for both margins was examined under a digital microscope (HiRoX, Tokyo, Japan) at 50X magnification to evaluate the microleakage and was blindly scored using the following scoring system [21, 22]:

- 0 = No dye penetration.
- 1 = Penetration not exceeding the middle of the cervical wall depth.
- 2 = Penetration exceeding the middle of the cervical wall depth.
- 3 = Penetration including the whole cervical wall but not including axial wall.
- 4 = Penetration including the axial wall.

The maximum score for each sample was considered for statistical analysis [23].

### Statistical analysis

The normality of the data was assessed by the Kolmogorov-Smirnov test to determine the statistical tests to be used (parametric vs. non-parametric).

Five out of eight variables produced significant p-values for those tests (*p* < 0.05) showing non-normal distribution. Hence, it was decided to use non-parametric tests for all the following analyses. Descriptive statistics such as median, variance, and minimum and maximum values were calculated for each subgroup. Non-parametric, Kruskal–Wallis tests were used for intergroup comparisons with more than two groups, while Mann–Whitney U-tests were performed for intragroup comparisons with two groups. The results were deemed statistically significant at *p* < 0.05, and all statistical analyses were performed using SPSS (IBM, version 22.0 SPSS Inc., Chicago, USA).

## Results

### Descriptive statistics

**Fig. 1:**
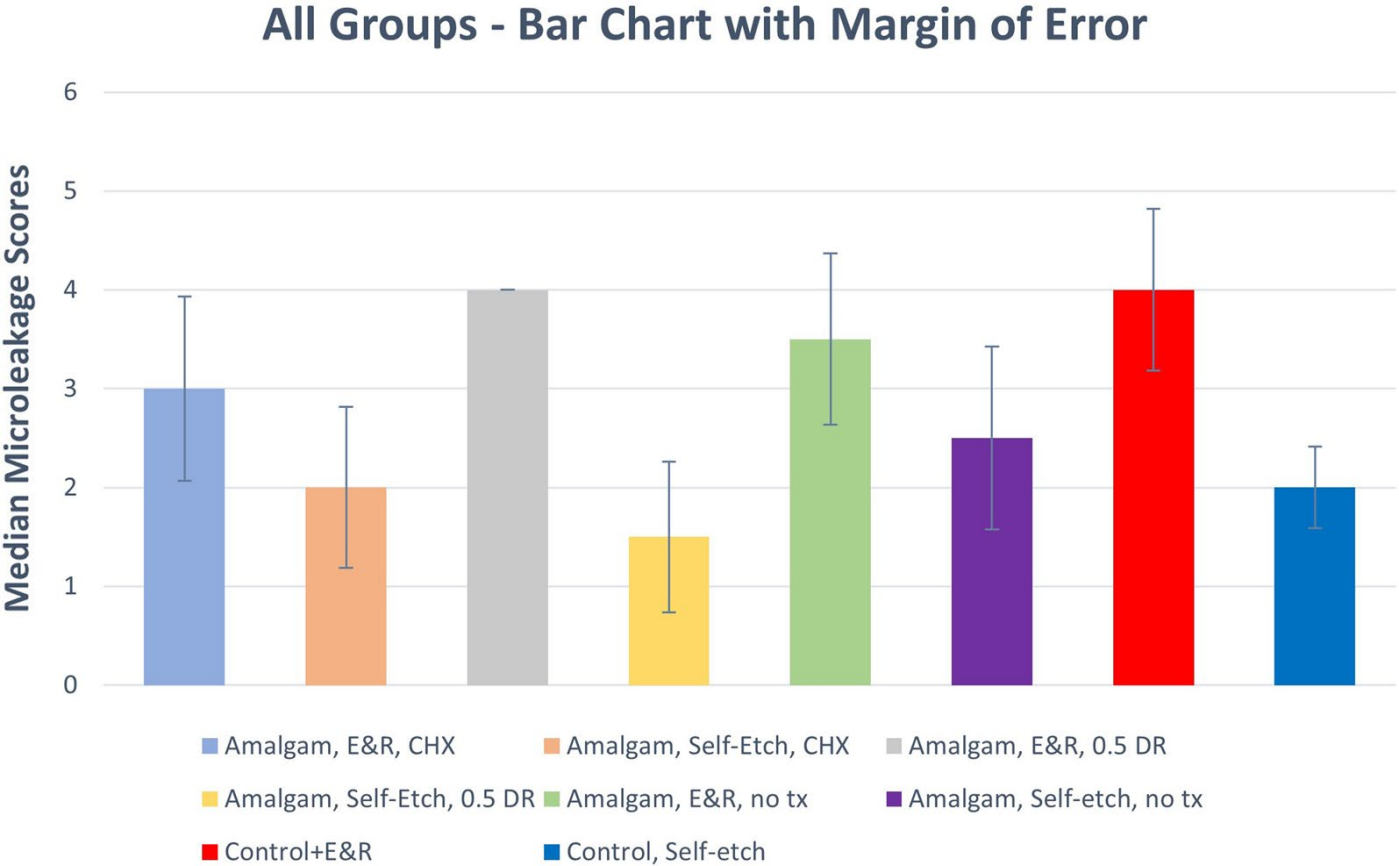
Median microleakage scores in all study groups, ER: etch-and-rinse. SE: self-etch. CHX: 2% chlorhexidine treatment. DR: dentin refreshment. NT: no treatment

**Fig. 2:**
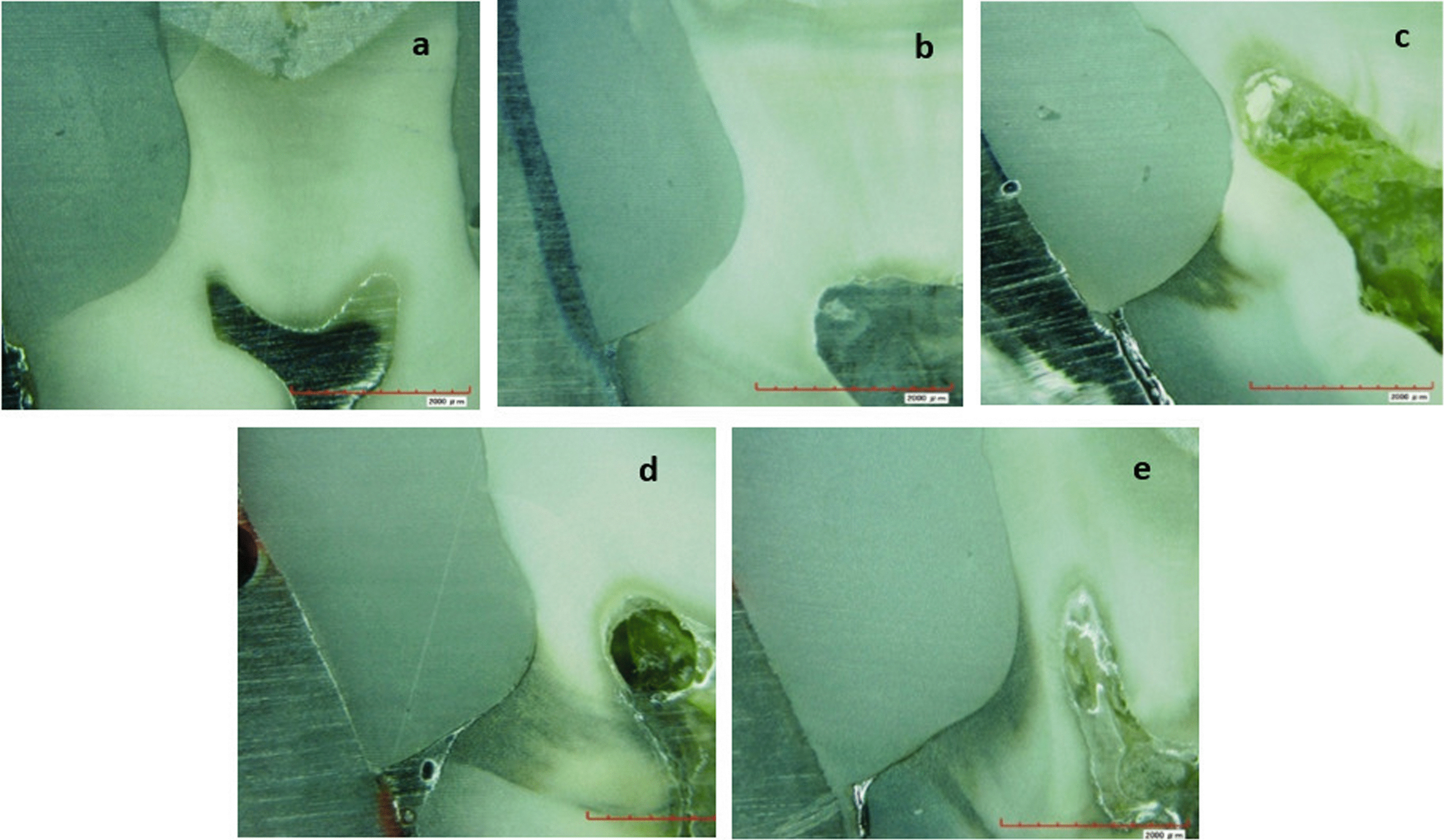
Photomicrographs representing different leakage scores. **a** CHX pretreated sample scored 0, **b** control sample scored 1, **c** no treatment sample scored 2, **d** and **e** control samples scored 3 and 4 respectively

Table 2; Fig. 1 presented the descriptive analysis for all tested groups. Microscopic images of different samples dye penetration scores are presented in Fig. 2. The highest microleakage scores were found in the dentin refreshment group (G2) when dentin acid-etching adhesive protocol was used. However, the lowest microleakage scores were found with the same group, G4, when the self-etching adhesive protocol was used. Generally, chlorhexidine group had lower microleakage scores, especially in the etch-and-rinse adhesive modes. Lower microleakage scores were observed for the no-treatment group in the self-etch adhesive protocol in comparison to other groups.

**Table 2:** Results of descriptive statistics and Kruskal–Wallis test for study groups

Descriptive statistics	Amalgam groups						Control groups	
	ER			SE				
	CHX	DR	NT	CHX	DR	NT	ER	SE
Median	3.00	4.00	3.50	2.00	1.50	2.50	4.00	2.00
Minimum	1	4	1	1	0	0	1	1
Maximum	4	4	4	4	4	4	4	3
*p*	0.065			0.845				

*ER* etch-and-rinse, *SE* self-etch, *CHX* 2% chlorhexidine treatment, *DR* dentin refreshment, *NT* no treatment

### Microleakage by adhesion protocols

All four groups (CHX, DR, NT, and control) within each adhesion protocol (ER and SE) were compared using two separate Kruskal–Wallis tests. No significant difference has been recorded between the four groups within each protocol[(*p* = 0.065, and *p* = 0.845) for the ER and SE protocols respectively] (Fig. 2; Table 2).

### Etch-and-rinse protocol

In teeth in which the etch-and-rinse adhesive mode was used, the highest leakage median values were found in the dentin refreshment (G2) and Control groups (G4) with a median score of 4.00 for both groups. In contrary, lower values were found in the no-treatment group (G3), with a median score of 3.50 while the lowest values were found in (G1) the chlorhexidine group (3.00). Nonetheless, differences within this protocol were not statistically significant (*p* > 0.05; Fig. 1; Table 2).

### Self-etch adhesion protocol

In teeth in which the self-etch adhesive protocol was used, the highest leakage scores were found in the no-treatment group (2.50) while the lowest values were found in the Dentin refreshment group (1.50). Chlorhexidine and control group scores were the same (2.0). Nonetheless, differences within this protocol were also not statistically significant (*p* > 0.05; Fig. 1; Table 2).

### Between-protocol comparisons

**Fig. 3:**
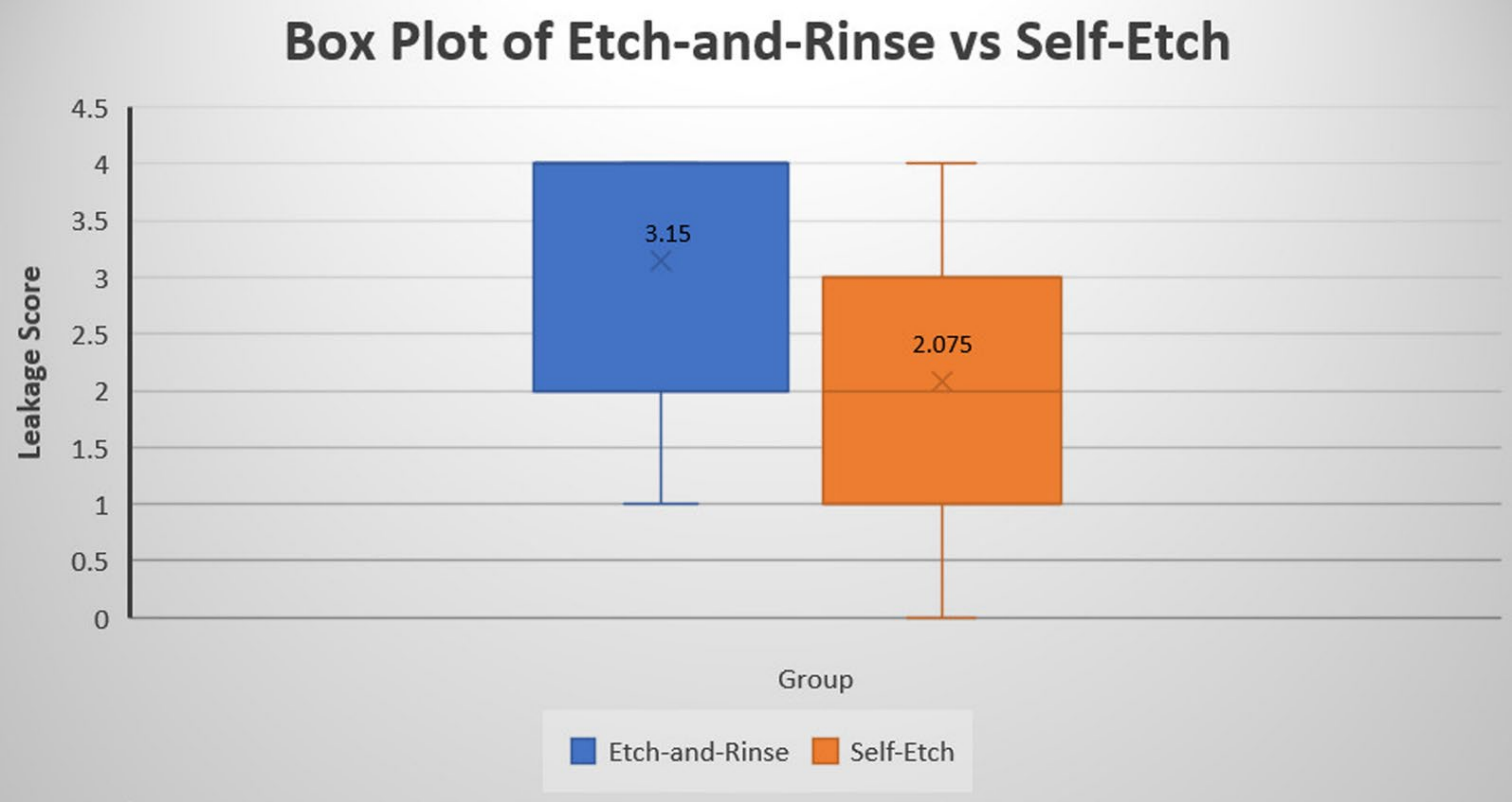
Boxplot illustrating the distribution of microleakage scores of Etch-and-Rinse versus Self-Etch groups

Data were pooled from ER and SE protocols independent of condition and tested for differences using a Mann–Whitney U-test. This analysis showed that, overall, leakage scores were significantly higher in the ER protocol as compared to the SE protocol (*p* < 0.001, Fig. 3; Table 3).

**Table 3:** Mann–Whitney U-test results for data comparison between the two adhesion protocols

Protocol	Median	Range	*p*-value
Etch-and-rinse	4	1–4	*p* < 0.001*
Self-etch	2	0–4	

*Statistically significant difference (*p* < 0.05)

### Microleakage by dentin pretreatment

When comparing respective conditions between protocols: e.g., CHX for the ER protocol with CHX for the SE protocol, using Mann–Whitney U-tests, significant differences were recorded for the control and DR conditions (*p*-values: 0.043 and < 0.001, respectively), indicating higher leakage for those conditions in the ER protocols as compared to the SE protocols (Table 4).

**Table 4:** Mann–Whitney U-tests for intragroup comparisons

Group	Median (IQR = Q3-Q1)	Range	*p*-value
*Control*			
Etch-and-rinse	4.00 (2.25)	1–4	*p* = 0.043*
Self-etch	2.00 (0.5)	1–3	
*CHX*			
Etch-and-rinse	3.00 (3)	1–4	*p* = 0.684
Self-etch	2.00 (3)	1–4	
*DR*			
Etch-and-rinse	4.00 (0)	4–4	*p* < 0.001*
Self-etch	1.50 (2)	0–4	
*NT*			
Etch-and-rinse	3.50 (3)	1–4	*p* = 0.436
Self-etch	2.50 (3)	0–4	

*ER* etch-and-rinse, *SE* self-etch, *CHX* 2% chlorhexidine treatment, *DR* dentin refreshment, *NT* no treatment

*Statistically significant difference (*p* < 0.05)

In general, Chlorhexidine pretreatment lowered microleakage scores in E&R adhesive protocol in comparison to the self-etch mode, but the difference was not significant (*p* > 0.05). The no-treatment produced higher values of microleakage in the E&R protocol compared to the self-etch protocol, but the difference was also not significant (*p* > 0.05).

## Discussion

The present study evaluated the effect of amalgam contamination, different surface treatments, and adhesive protocols on dentin microleakage to bulk-fill composite resin material. Based on the results of this study, the microleakage of non-contaminated dentin was not significantly different compared to the amalgam-contaminated dentin groups irrespective of treatment within each protocol; therefore, the null hypothesis that there would be no significant difference between amalgam- and non-contaminated dentin bonded to bulk-fill composite using different surface treatments was accepted. Additionally, dentin treatment in general affected the microleakage in all groups pooled together but specifically in chlorhexidine and dentin refreshment; therefore, the second hypothesis was that there would be no significant difference in microleakage scores of different protocols of the universal adhesive system was partially rejected.

Silver nitrate is one of the most commonly used dye tracers for microleakage evaluation and can be used with different micro-investigative techniques [24]. It provides good contrast during the microscopic examination and has the ability to be immobilized during sectioning and examination [25]. Ammoniacal silver nitrate was used in place of the conventional acidic silver nitrate to minimize the demineralizing effect [26]. It also has a high affinity to bind to exposed collagen fibrils that are not covered by resin monomers [27].

To prevent underestimation of leakage scored, three sections were made per sample and the highest reading was recorded as the final score for the sample [28].

In this study, the self-etching adhesive protocol was found to significantly reduce dentin microleakage compared to the etch-and-rinse adhesive protocol. This can be observed clearly in the control group where no treatment or corrosive products were present. In the self-etch group, the removal of 0.5 mm dentin had comparable results to those of the control group.

These results emphasize that the use of universal adhesive containing 10-methacryloyloxydecyl dihydrogen phosphate (MDP) may be preferable in deep class II or V dentin or where little or no enamel is present. This comes in agreement with many studies which indicate that the use of universal adhesive in self-etch mode reduces microleakage at the dentin resin interface better than the etch-and-rinse protocol [29–31].

The work of Muñoz et al. [29] compared the nanoleakage of universal adhesives containing the MDP monomer including ScotchBond Universal to other adhesive systems lacking this monomer. They found that universal adhesives containing the MDP monomer had stable bonds over time. In addition, the presence of the MDP monomer and the Vitrebond copolymer gave the advantage of having a more stable dentin-resin interface over other self-etch adhesives containing only the MDP monomer.

On the other hand, Kermanshah et al. [30] compared the microleakage of enamel and dentin margins using ScotchBond universal in self-etch and etch-and-rinse modes. They found that less leakage was observed in dentin margins when using the self-etch protocol compared to the etch-and-rinse mode while the opposite is true for the enamel margins.

Moreover, Tran et al. [31] compared several etch-and-rinse with self-etch adhesives containing different functional monomers. They concluded that, of the two, the self-etch adhesives showed better long-term adhesive-dentin interface stability. In addition, the thicker hybrid layer found in the etch-and-rinse systems did not equate to the more stable bond interface.

This may be explained by ionically interaction of 10-MDP monomer with the abundantly present hydroxyapatite around collagen fibrils to form MDP-Ca salts nanolayers, which are stable and water insoluble [32, 33].

Moreover, this mild form of self-etching monomer causes less dissolution of smear plugs and less opening of dentinal tubules, therefore reducing dentin permeability. The functional monomer mild acidity facilitates etching while preserving hydroxyapatite, penetration, and impregnation of other monomers, therefore creating a relatively thick hybrid layer, which might also contribute to reduced microleakage [34].

In this study, it can be observed that the chlorhexidine treatment had a positive effect in terms of reducing microleakage, especially in the etch-and-rinse group.

It is well documented that acid etching will cause the dissolution of the inorganic component of dentin that exposes collagen fibrils and can cause latent forms of MMP to be activated [35]. It was found that both the two-step etch-and-rinse and the one-step self-etch adhesives can activate MMP-2 and MMP-9. Nevertheless, higher activity was found to be associated with etch-and-rinse adhesives in which greater exposure of dentin matrix occurs [36].

The use of MMP inhibitors such as chlorhexidine digluconate can stabilize the adhesive layer and prevent its degradation over time. This is especially true for the etch-and-rinse adhesive systems but is still controversial for self-etch adhesives [12, 37].

This difference was evident in our study where chlorhexidine treatment did not affect microleakage negatively. In fact, it reduced the microleakage in the etch-and-rinse group in comparison to the control and the 0.5 mm reduction group but was not significantly different from the no-treatment group. This comes in agreement with Saffarpour et al. [38], who found that chlorhexidine application in the etch-and-rinse group, without rinsing chlorhexidine, significantly reduced microleakage in the dentin margin.

An explanation to why the two-step etch-and-rinse systems could benefit more from chlorhexidine pre-treatment than some self-etch adhesives is that the application of disinfectant may leave dentin more resistant to acid conditioning. This might be clearer with adhesives that use weak acids to demineralize dentin [39].

It is also noted that the unmodified group, especially in the etch-and-rinse mode, had lower microleakage scores than those in the modified or control groups. This comes in agreement with Sabarathinam et al. [40], Alptekin et al. [41], and Patel et al. [42], who compared microleakage of composite resin to amalgam restorations using different microleakage methods. They all found that amalgam restorations had lower microleakage than composite restorations.

This difference might be explained by the formation of corrosion products underneath the amalgam restorations sealing the tooth restoration margin [43]. Many elements such as zinc, silver, tin, and copper can be found in dentinal tubules [44]. Moreover, corrosion byproducts such as silver sulfides attach to collagen fibrils making the etching process more resistible [45, 46].

This difference can be manifested in the etch-and-rinse group, where the no-treatment group had the lowest microleakage score. This can be explained by the presence of corrosive products or the resistance to the etching process, which therefore will reduce the effect of MMP activity.

In the self-etch adhesive protocol, the highest score of microleakage was reported in the no-treatment group as compared to the control, 0.5 mm dentin removal, or CHX groups. This indicates that the use of mild self-etchant with the already resistant substrate may negatively impact the results. Nonetheless, the use of the self-etch protocol produced a lower microleakage score than did the etch-and-rinse protocol. It is noted that in the etch-and-rinse adhesive protocol, the worst leakage score was found in the dentin refreshment treatment. This might be attributed to the changes in dentin structure and permeability as the depth increases. Deeper parts of dentin have wider dentinal tubules which make fluid influx easier, in addition to the effect of acid etching [47]. This can result in a more difficult bonding procedure, especially for the hydrophobic resin monomers [48].

In a study by Redwan et al. [49], the microleakage of composite resin restorations in freshly cut dentin were compared to the group of replaced amalgam restorations. They used thirty extracted human molars, which they restored with class II high Copper amalgam restorations, then thermocycled for 10,000 cycles. After that, they used 25 teeth to replace the amalgam with composite resin restorations. An opposite class II was made in each tooth and restored with composite resin in freshly cut dentin using the same dimensions. They selected twenty teeth for microleakage testing using silver nitrate. The authors did not find any significant difference between the two groups. Therefore, they indicated that corrosion products were not found in the dentin of replaced amalgam. Nonetheless, the cavity design in Redwan et al.’s study was different than this study, as they included enamel cervical margins (1 mm above CEJ). Moreover, the adhesive system used was different (ExciTE F DSC, Ivoclar Vivadent, Amherst, NY, USA).

In Ghavamnasiri et al. study [50], the microleakage of dentin restored with composite resin after amalgam replacement has been investigated. The authors created class II cavities in premolars and divided the teeth into four groups. The first group was restored with conventional composite. The second group was restored with an admixed high copper content amalgam after applying a varnish. The amalgam restored specimens were kept for six months in normal saline at 37^o^ degrees Celsius. The third group was restored in the same method, but the amalgam was replaced with composite. For the fourth group, the cavity was extended 0.5 mm beyond the original cavity. They indicated that amalgam contaminated group had higher leakage scores than control and that 0.5 mm dentin refreshment had similar leakage scores as the control. The authors reported that leakage could occur because amalgam corrosion products could prevent the full monomer infiltration or reduce the acid solubility of smear layer, and therefore, preventing its removal. The adhesive used in their study was one-step (Bisco, Chicago, Schaumburg, Illinois, USA) in etch-and-rinse adhesive protocol. In contrary, the results of the current study, they have demonstrated high leakage scores in the amalgam contaminated group. The difference in adhesive compositions and the way in which the hybrid layer forms as a result, may partially explain the contradiction between the two studies.

## Limitations of the study

Marginal integrity tests are not the best factors to determine the impact of using different materials, surface treatments, or adhesion protocols in clinical dentistry [51]. Microleakage studies might seem to be more relevant to clinical situations than bond strength for the assessment of bond durability over time. However, the reliability of conventional two-dimensional studies remains controversial [52].

In addition to the great variability in microleakage studies and the methodology used precludes systematic review analysis, the characteristics of the molecular dyes used in these tests are not specific. Therefore, such laboratory studies should be correlated to clinical measures assessment for validation [53].

One of the limitations of this study is the relatively small sample size. Increasing the sample size may reflect a more reliable result. Also, dye penetration tests can be replaced with micro computed tomography method, whenever possible, which precludes the destruction of the sample used. Moreover, only one bulk fill resin composite material was used in this study, using different types of dental composites, like other bulk fill composites and conventional resin composite materials will add more value to the results of the study.

## Conclusions

Within the limitations of this study, it can be concluded that amalgam contamination did not affect dentin microleakage. However, Chlorhexidine pretreatment had a positive effect by reducing microleakage in both adhesive protocols. The effect of the adhesive protocol was dominant, favoring the self-etch mode over the etch-and-rinse mode despite the dentin condition.

## Data Availability

The datasets used and/or analyzed during the current study are available from the corresponding author on reasonable request.

## Abbreviations

ER: Etch-and-rinse
SE: Self-etch
CEJ: Cemento-enamel junction
CHX: Chlorhexidine
DR: Dentin refreshment
NT: No treatment
NaOCL: Sodium hypochlorite
MDP: 10-Methacryloyloxydecyl dihydrogen phosphate
MMP: Matrix metalloproteinase
